# Surface force analysis of glycine adsorption on different crystal surfaces of titanium dioxide (TiO_2_)

**DOI:** 10.1186/s40580-017-0125-y

**Published:** 2017-12-08

**Authors:** Narangerel Ganbaatar, Kanae Imai, Taka-aki Yano, Masahiko Hara

**Affiliations:** 1 Chemical Evolution Lab Unit, Earth-Life Science Institute (ELSI), Tokyo Institute of Technology, Tokyo, Japan; 2 Department of Electronic Chemistry, Interdisciplinary Graduate School of Science and Engineering, Tokyo Institute of Technology, Yokohama, Japan; 3 Department of Chemical Science and Engineering, School of Materials and Chemical Technology, Tokyo Institute of Technology, Yokohama, Japan

**Keywords:** Atomic force microscopy (AFM), Titanium dioxide (TiO_2_), Glycine, Chemical evolution, Origins of life

## Abstract

Surface force analysis with atomic force microscope (AFM) in which a single amino acid residue was mounted on the tip apex of AFM probe was carried out for the first time at the molecular level on titanium dioxide (TiO_2_) as a representative mineral surface for prebiotic chemical evolution reactions. The force analyses on surfaces with three different crystal orientations revealed that the TiO_2_ (110) surface has unique characteristics for adsorbing glycine molecules showing different features compared to those on TiO_2_ (001) and (100). To examine this difference, we investigated thermal desorption spectroscopy (TDS) and the interaction between the PEG cross-linker and the three TiO_2_ surfaces. Our data suggest that the different single crystal surfaces would provide different chemical evolution field for amino acid molecules.

## Introduction

Many organic molecules are chemically or physically adsorbed to mineral surfaces in aqueous solutions. The extent of adsorption is dependent on variables including mineral type, surface roughness, type of adsorbed organic molecule, and the nature of the solvent with respect to properties such as pH, ionic strength, temperature, and the concentration of dissolved ions, which may act as mediators for binding [1, 2]. Among all mineral surfaces, titanium dioxide (TiO_2_) is well known for its ability to adsorb biomolecules. Understanding the interaction of biomolecules with TiO_2_ surfaces is important for many areas of modern technology, including photocatalysis, dye-sensitized solar cells, and biological implants used in dental and orthopedic applications [3, 4]. Regarding research on origins of life, the surfaces of this mineral have long been studied as possible catalysts for small organic compound reactions and considered as a host inorganic catalyst in prebiotic evolution studies. Moreover, rutile TiO_2_ is one of the minerals that have been discovered as micro- or nanoparticles in pre-solar dust grains (also known as interplanetary dust particles) and is included in the “mineral evolution” hypothesis, which outlines 10 stages of near-surface mineral diversification since the formation of the Earth [5]. However, not much is known about how TiO_2_ surfaces interact with biomolecules at the molecular level. Understanding the fundamental mechanisms underlying the adhesion of biomolecules on TiO_2_ surfaces is essential to the identification and improvement of materials suitable for medical applications. Therefore, spectroscopic analyses are required for assessing these mechanisms.

Previous theoretical studies of the interaction of amino acids with TiO_2_ surfaces, which were based on the density functional theory (DFT), have contributed to understanding the main adsorption modes and are used as prototypes to study protein-surface interactions. Recently, Sowmiya and Senthilkumar used first-principle calculations to determine that anatase TiO_2_ crystals with more (001) facets adsorb proline, hydroxyproline, and glycine, i.e., major components of the collagen protein. According to their results, amino acids strongly bind to the anatase TiO_2_ (001) surface in the dissociative adsorption configuration mainly because of the covalent interaction between the oxygen atoms of the amino acid and the titanium atoms on the mineral’s surface [6]. Tillotson et al. used DFT calculations to study how small organic molecules interact with the TiO_2_ (110) surface. According to their calculations, glycine strongly binds to the Ti*5f* ion either via the amino (–NH_2_) or the carboxyl group (–COOH). However, the most stable binding mode for glycine occurred via the interaction of the two oxygen atoms of the carboxylic group oxygen with two Ti*5f* sites. The proposed reaction involves the transfer of a proton to the surface forming a hydroxyl group and is likely to also apply to larger molecules containing the COOH group [7]. Moreover, the DFT calculations of Tonner demonstrated a probable tilting stabilization effect between the surface-bound hydrogen atom and the nitrogen atom of the amino group, suggesting that van der Waals interactions stabilize the tilted configurations even further by reinforcing the attraction between the amino group and the surface [8].

Besides these theoretical studies, lots of experimental investigations have also been performed. Examples of these studies include the proline adsorption on TiO_2_ (110) and (011) single crystal surfaces that was demonstrated by synchrotron-based X-ray photoemission spectroscopy (XPS) and temperature programmed desorption (TPD) [9–11], and the adsorption of glycine on TiO_2_ (110) single crystals that was detected by synchrotron-based ultraviolet photoemission spectroscopy (UPS) [12, 13], scanning tunneling microscopy (STM) [14], and plane-wave DFT calculations [15].

However, as far as we know, there are no force studies of the adsorption of amino acids on the TiO_2_ surface at the molecular level. Therefore, we decided to study the adsorption of glycine, which is the amino acid with the simplest structure, on TiO_2_ surfaces by performing AFM force measurements.

### Surface structures of TiO_2_ (110), (100), and (001) for surface force analysis

TiO_2_ is typically thought of as being chemically inert and as the most active photocatalyst found in nature, using the energy of light to promote chemical reactions. Importantly, TiO_2_ is an active catalyst for carbon–carbon bond formation reactions in which higher-molecular-weight compounds are synthesized from smaller ones [16].

TiO_2_ crystallizes in three major different structures: rutile (tetragonal, a = b = 4.584 Å, c = 2.958 Å), anatase (tetragonal, a = b = 3.782 Å, c = 9.514 Å), and brookite (rhombohedrical, a = 5.436 Å, b = 9.166 Å, c = 5.135 Å) [16].

**Fig. 1:**
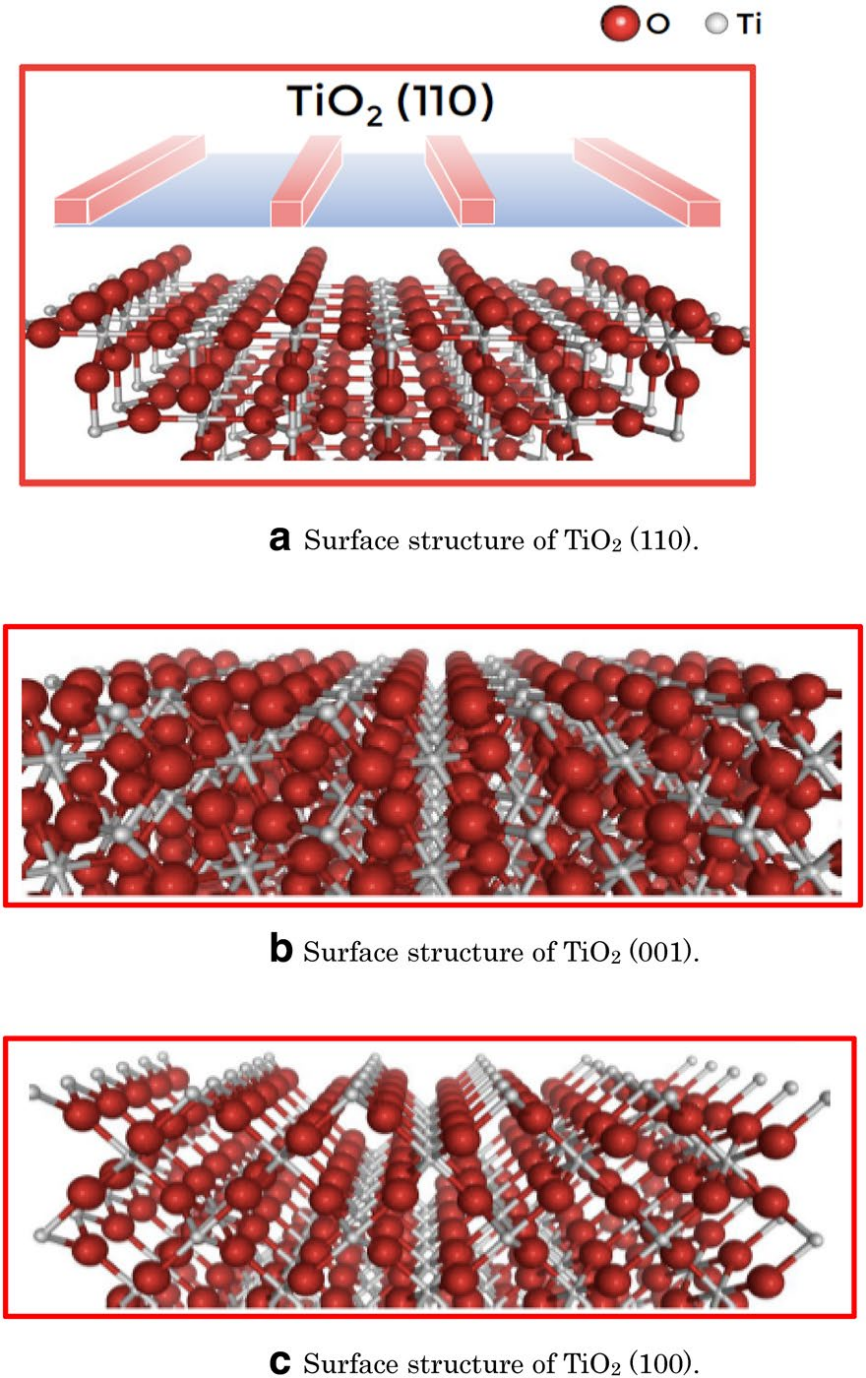
**a** Surface structure of TiO_2_ (110). **b** Surface structure of TiO_2_ (001). **c** Surface structure of TiO_2_ (100)

The rutile TiO_2_ (110) is known to be one of the most stable TiO_2_ crystal faces, while rutile and anatase are used in TiO_2_ applications and are of interest for surface science studies (Fig. 1a–c). Its surface contains two different kinds of titanium atoms. Along the (001) direction, rows of sixfold-coordinated Ti atoms alternate with fivefold-coordinated Ti atoms with one dangling bond that is perpendicular to the surface. Two kinds of oxygen atoms are created when the bridging oxygen atoms miss one bond to the Ti atom in the removed layer and are twofold coordinated [16]. These bridging oxygen atoms and the precise structure of the surface have been the subject of debate for many years. Because of their coordinative undersaturation, atoms from these rows are thought to be removed relatively easily by thermal annealing. The resulting point defects affect the overall chemistry of the surface [17].

On the (001) surface, all Ti atoms are fourfold-coordinated and all O atoms twofold-coordinated. Hence, it has been proposed that the number of broken bonds on this surface is higher than on the other low-index rutile surfaces discussed so far. Consequently, the (001) surface has a high surface energy and tends to facet or reconstruct. This surface is the least studied with respect to structural information [16].

The rutile TiO_2_ (100) surface has received considerably less attention than the (110) crystal face. Some research groups have conducted several theoretical calculations for this crystal surface. In general, all these calculations and theoretical approaches agree that Ti atoms are fivefold-coordinated on the (100) face.

Perron et al. carried out calculations on the different crystal structures of rutile TiO_2_ surfaces and computed rutile phases, structures, relaxations, and surface energies of the (110), (100), (101), and (001) faces. They found the (110) face to be the most stable one, followed by (100) and (101) [18, 19]. Additional theoretical and experimental work aimed at resolving the geometry of this surface would be quite valuable.

## Experimental procedures

### Sample preparations (TiO_2_ substrates)

The (110), (100), and (001) TiO_2_ surfaces were used to study the interactions with glycine. Commercially available rutile TiO_2_ substrates of all crystal planes were bought from Shinkosha (Tokyo, Japan).

New substrates were not cleaned by any further treatment and were directly used for AFM measurements. Used substrates were cleaned with deionized water and ethanol in ultrasonication to remove any contaminants from their surface.

### Attaching glycine molecule to the AFM cantilever tip

Glycine (NH_2_CH_2_COOH) molecules were anchored on an AFM tip apex to study the interactions of this amino acid with TiO_2_ (110), (100), and (001) surfaces. This linear molecule consists of three groups and two configurations are possible, as it is neutral in gas and zwitterionic in solid phases.

Moreover, glycine is the smallest of the amino acids. It is ambivalent, meaning that it can be inside or outside of the protein molecule. In aqueous solution and at, or near, neutral pH, glycine exists predominantly in the zwitterion form.

With a well-established tip-modification method as previously reported [20], glycine molecules were introduced to the AFM tips. Commercial soft cantilevers (OMCL-TR400 PB-1, with both sides gold-coated) with nominal spring constants of 0.02 N/m were used. Cantilevers were cleaned in UV ozone cleaner and followed by washing with ethanol and deionized water to be ready for the further modification steps. The cantilevers were first introduced in a mixture of two types of thiol reagents in ethanol (2 mM 1,8-octanedithiol and 20 mM 6-mercapto-1-hexanol). Mixed solutions were used to increase the probability of single-molecule events by controlling the densities of molecules on the tips as well as to prevent aggregation on the tip surfaces. The cantilevers were inserted into the solution for more than 18 h to introduce the thiol groups. The modified cantilevers with thiol groups were washed with ethanol. MAL-PEG-NHS cross-linker molecules were anchored to the tips by stable covalent bonds formed during the reaction of their maleimide ends with the –SH groups of alkanethiols. The thiol activated cantilevers were incubated for 60 min with 1 mg/ml of NHS-PEG-MAL in toluene. They were then washed several times with PBS to remove unreacted crosslinkers. Finally, glycine residues were cross-linked to the tips through the free NHS ends of the PEG cross-linker molecules by inserting the cantilevers into the solution for 2 h.

## Results

### AFM force measurements

In the single-molecule force spectroscopy measurements, several hundred force–distance (F–D) curves were recorded with at least two glycine-modified AFM tips at room temperature in a phosphate-buffered saline (PBS) solution (pH 7.4) to minimize the nonspecific interactions between the probe and the substrate. By lowering each modified AFM tip onto the measured surface to a maximum load of 400 pN at a loading rate of 6 nN/s, typical F–D curves were obtained from the contact between each tip and the three different crystal faces of TiO_2_ surfaces.

**Fig. 2:**
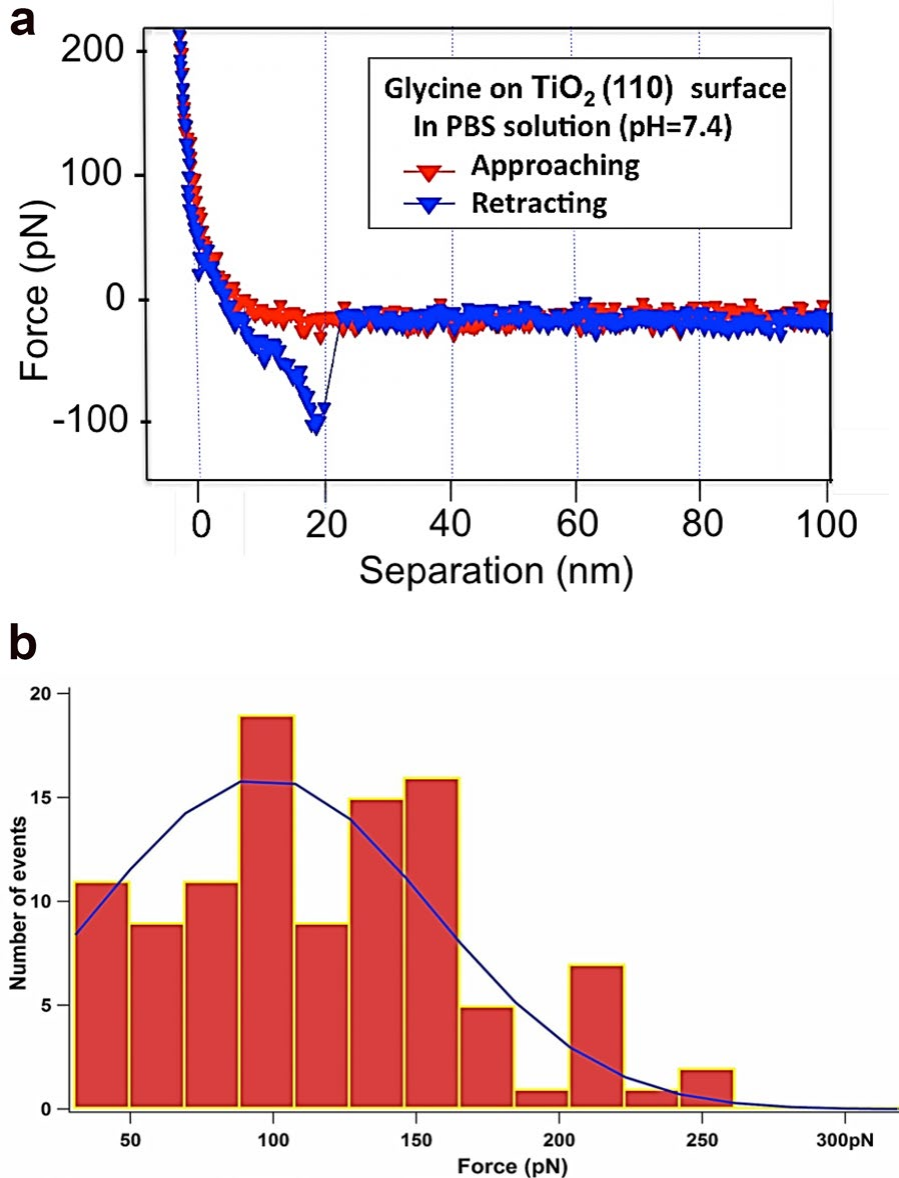
**a** Specific adhesion curve obtained on TiO_2_ (110) surface. **b** Unbinding force histogram. MPF for glycine on TiO_2_ (110) surface at a loading rate of 6 nN/s (pH 7.4). Fitting was performed with a Gaussian fit and shows a mean force of 128.31 ± 38.88 pN

Glycine showed a specific interaction with the TiO_2_ (110) surface (Fig. 2a). The extension length (i.e., the length at the maximum physically possible extension of the cross-linker polymer) ranges from ~ 10 to ~ 20 nm. This range is considerable given the total length of molecules that were used to mount the glycine molecules on the tips. However, the fact that molecules can exist in different conformations may have contributed to the overall variety of the extension lengths. The cross-linker, NHS-PEG-MAL, has a standard extended length of ~ 10 nm while the preceding short thiol molecules are ~ 2 nm long. Since the molecular length of the glycine is very small, most of the extension must have resulted from the linker molecules, which could have been extended to a total length of approximately 12–15 nm. In addition, the adhesion forces recorded in these experiments ranged from ~ 90 to 150 pN and the probability of adhesion was low (8 ~ 10%).

A histogram was constructed for the number of adhesion events recorded on TiO_2_ (110) surface (total number of events: n ≈ 60) versus rupture force and was used to obtain the most probable force (MPF), which was 128.31 ± 38.88 pN (Fig. 2b). Only the rupture events that occurred when the PEG linkers were extended by ~ 10 to ~ 20 nm are shown in the given histograms.

**Fig. 3:**
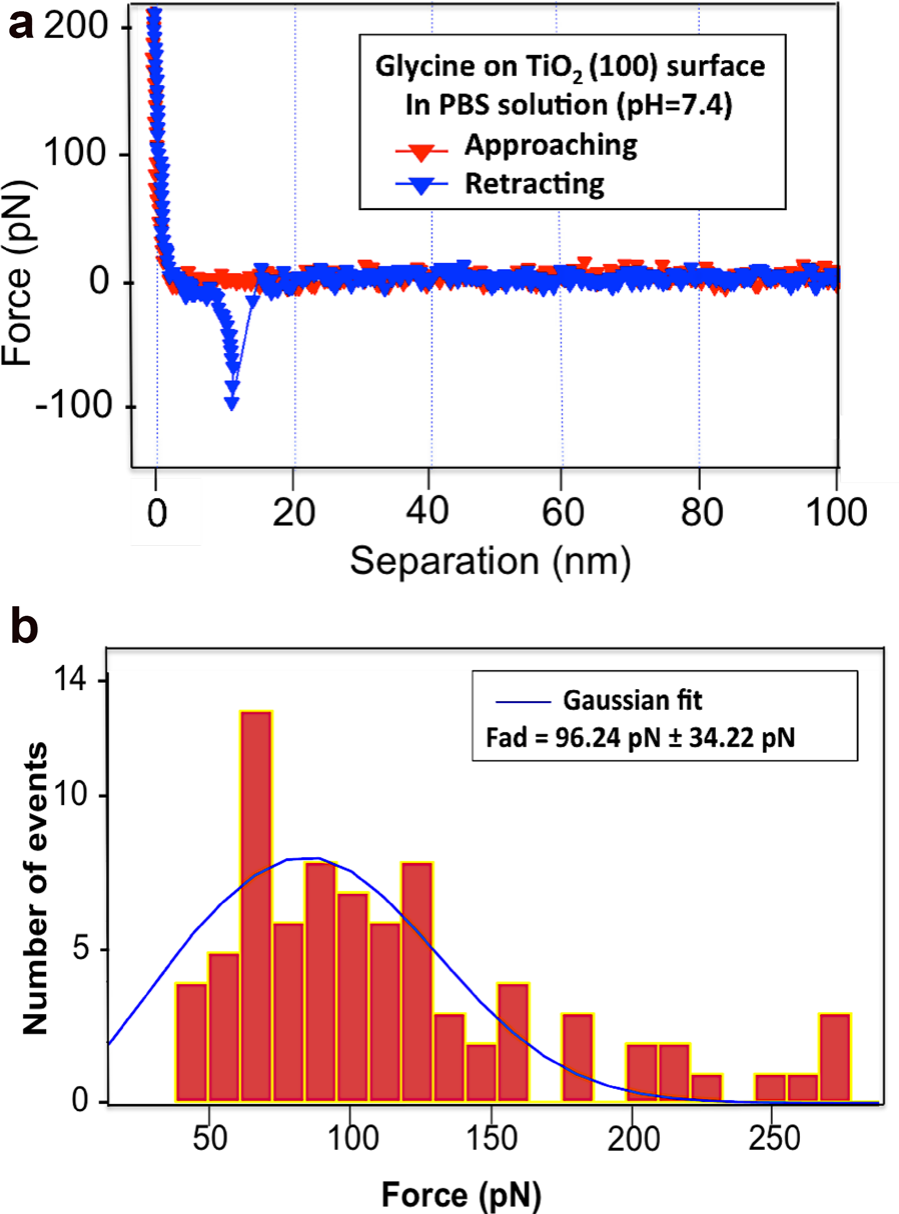
**a** Specific adhesion curve obtained on TiO_2_ (100) surface. **b** Unbinding force histogram. MPF for glycine on TiO_2_ (100) surface at a loading rate of 6 nN/s (pH 7.4). Fitting was performed with a Gaussian fit and showed a mean force of 96.24 ± 34.22 pN

Specific adhesion was also observed in the force measurements of glycine on TiO_2_ (100) surface (Fig. 3a). The extension length was considerable and ranged from ~ 10 to ~ 20 nm. The adhesion forces recorded in these experiments ranged from ~ 60 to 120 pN, i.e., weaker than those recorded on the TiO_2_ (110) surface.

The histogram was built by collecting the appropriate force curves (total number of events: n ≈ 60) and the Gaussian fit indicated an MPF of 96.24 ± 34.22 pN (Fig. 3b). Only those rupture events that occurred when the PEG linkers were extended by ~ 10 to ~ 20 nm are shown in the histogram.

**Fig. 4:**
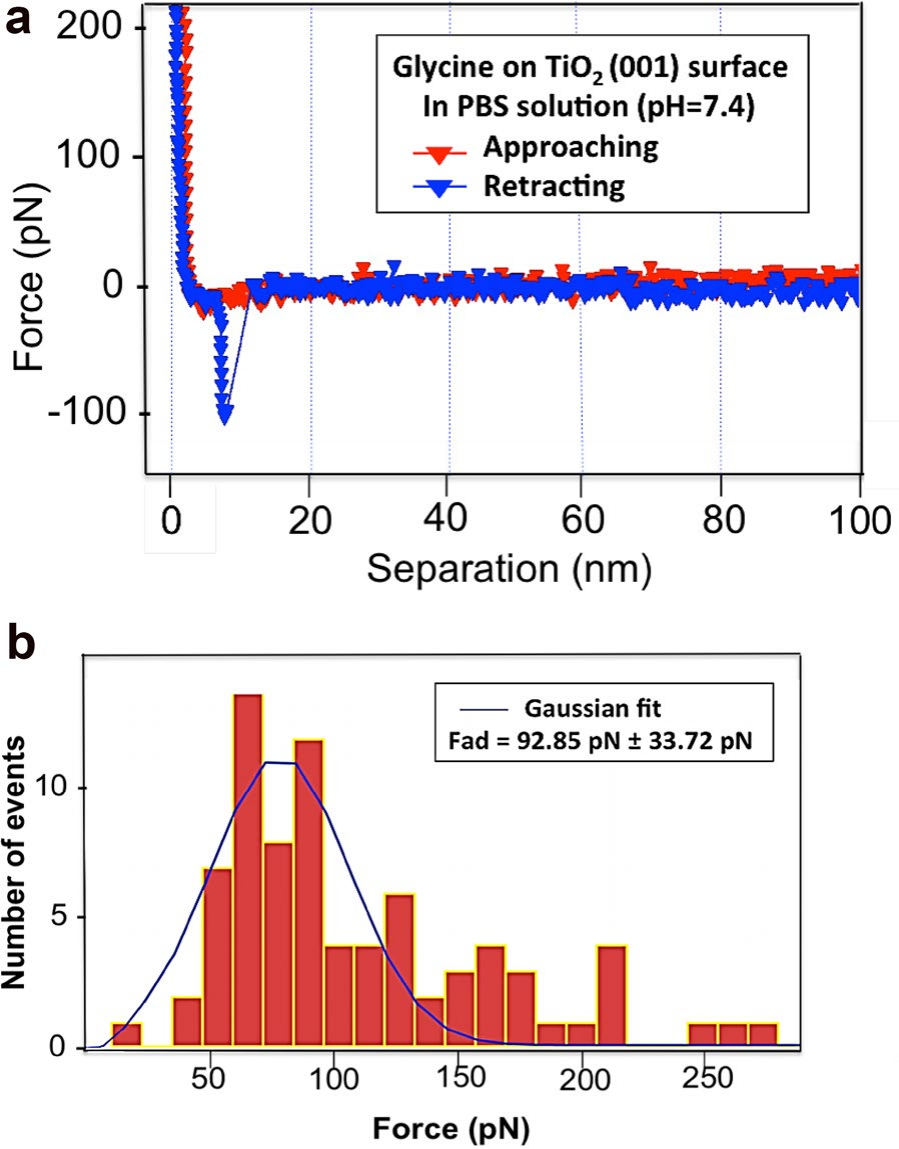
**a** Specific adhesion curve obtained on TiO_2_ (001) surface. **b** Unbinding force histogram. MPF for glycine on TiO_2_ (001) surface at a loading rate of 6 nN/s (pH 7.4). Fitting was performed using a Gaussian fit and showed a mean force of 92.85 ± 33.72 pN

Glycine also showed a specific interaction with the TiO_2_ (001) surface (Fig. 4a). There was no significant difference in adhesion forces between glycine and the TiO_2_ (001) surface compared to the TiO_2_ (100) surface. Moreover, the resulting histogram indicated a similar MPF value (92.85 ± 33.72 pN) (Fig. 4b). The adhesion force was higher on the TiO_2_ (110) surface (128.31 ± 38.88 pN), compared to the other two surfaces.

### Thermal desorption spectroscopy (TDS) measurements

**Fig. 5:**
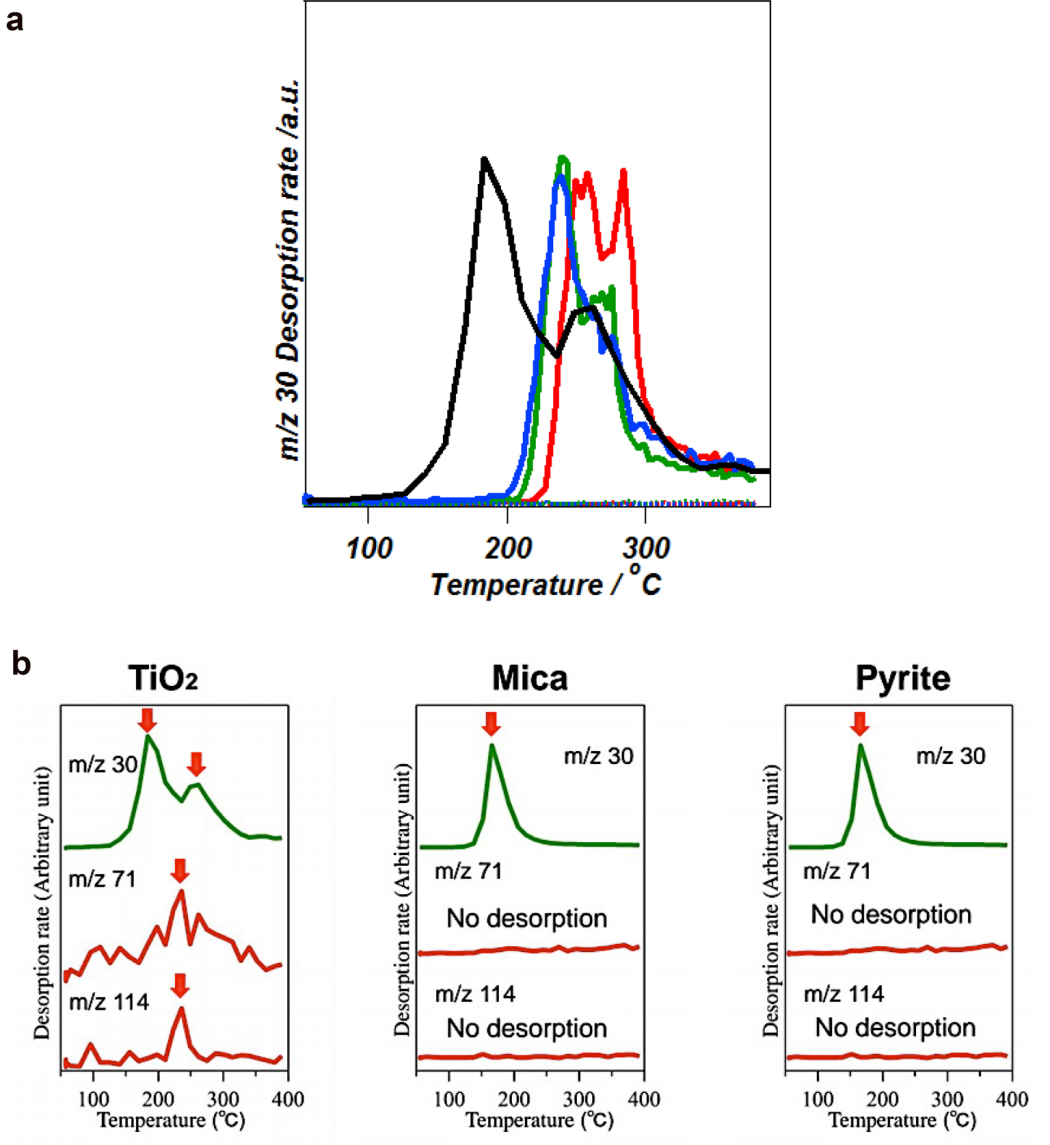
**a** Desorption of glycine from single crystals of TiO_2_ (110), (100), and (001). Polycrystalline was included as a reference substrate. Red line = TiO_2_ (110), green line = TiO_2_ (100), blue line = TiO_2_ (001), and black line = polycrystalline. **b** Comparison of interactions between glycine and TiO_2_, mica, or pyrite by TDS measurements

Thermal Desorption Spectroscopy (TDS) measurements were conducted to check the glycine desorption on all three TiO_2_ (110), (100), and (001) crystal plane surfaces. All surfaces displayed a desorption peak at m/z 30 indicating the desorption of glycine monomers. With respect to temperature, desorption at m/z 30 had sharp peaks at 260–285 °C in the case of TiO_2_ (110), at 240 °C in TiO_2_ (100), and at 240 °C in TiO_2_ (001) (Fig. 5a). These results suggest that, at a higher temperature, TiO_2_ (110) interacts with glycine more strongly than with other crystal faces. This is consistent with the AFM force measurements in which the adhesion force was higher for the TiO_2_ (110) surface compared to the other two surfaces.

TDS measurements were also carried out on mica and pyrite for comparison with TiO_2_ (Fig. 5b). The maximum desorption temperature for the glycine monomer (m/z 30) was 200 °C for TiO_2_ and around 150 °C for mica and pyrite. Importantly, TiO_2_ also displayed desorption peaks at m/z 71 and m/z 114, corresponding to glycine dimers with a maximum at 245 °C. The fact that, among the three kinds of surfaces, polymerization of glycine was only observed on TiO_2_, suggests that TiO_2_ plays an important role in the polymerization reaction of glycine.

Overall, the TDS results revealed the factors because of which glycine interacts more strongly with TiO_2_ compared to the other minerals. Interaction is mainly categorized into two types: physical and chemical adsorption. Physical adsorption is weak and results from van der Waals forces or electrical interactions. Chemical adsorption is strong as electron exchange takes place between the adsorbed molecule and the surface, resulting in the formation of bonds (covalent, ionic, metal, or coordinate). These results were in agreement with the force measurements in which glycine showed specific interaction with TiO_2_ but not pyrite or mica surfaces.

## Discussion

### Difference in force curves

**Fig. 6:**
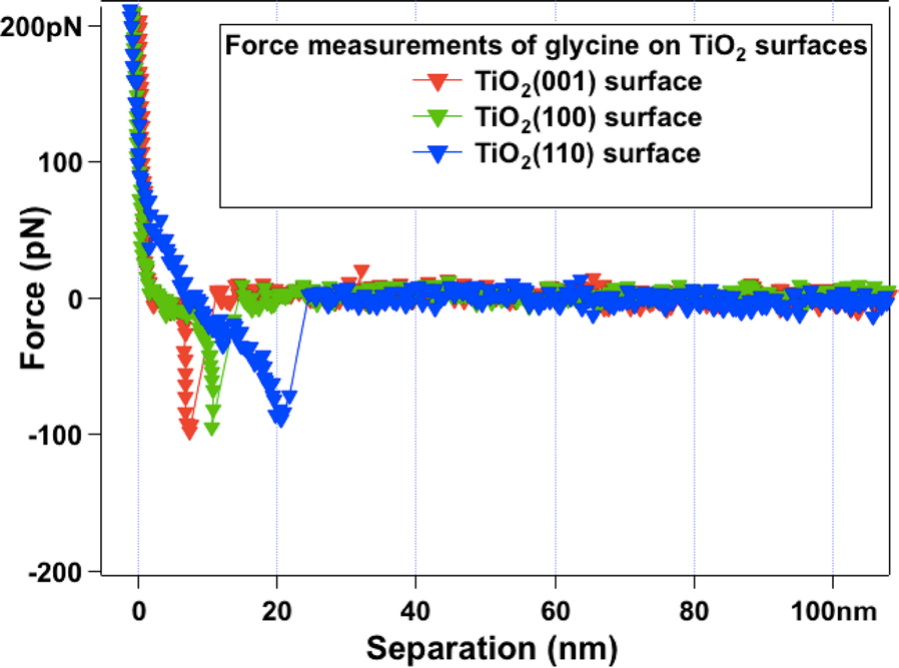
Force measurements between a glycine-modified tip and TiO_2_ (110), (100), and (001) surfaces

Even though all TiO_2_ crystal surfaces showed specific adhesion towards the glycine-modified AFM tips, differences were observed regarding the shape of the force curves. There was not a significant difference between the F–D curves obtained on TiO_2_ (100) and TiO_2_ (001) surfaces, but the shape of adhesion curves at TiO_2_ (110) was very different compared to the F–D curves obtained at the other two surfaces (Fig. 6a). In the case of TiO_2_ (110), the attraction began after the tip and the surface had already been in contact, resulting in a force curve with a broader shape. It should be noted that the same modified AFM tips were used on all three substrates, while the substrates were measured in the following order: TiO_2_ (110), (100), and (001). Thus, the difference in the shape of the curves cannot be attributed to contamination. We made the hypothesis that an interaction between the PEG linker and the surface taking place before the adhesion of the glycine molecules to the TiO_2_ (110) surface may be responsible for the difference in curve shapes. To test this hypothesis, we conducted experiments to check the interaction between the PEG molecules and the TiO_2_ surfaces.

### Possible interaction between the PEG linker and TiO_2_ surfaces

**Fig. 7:**
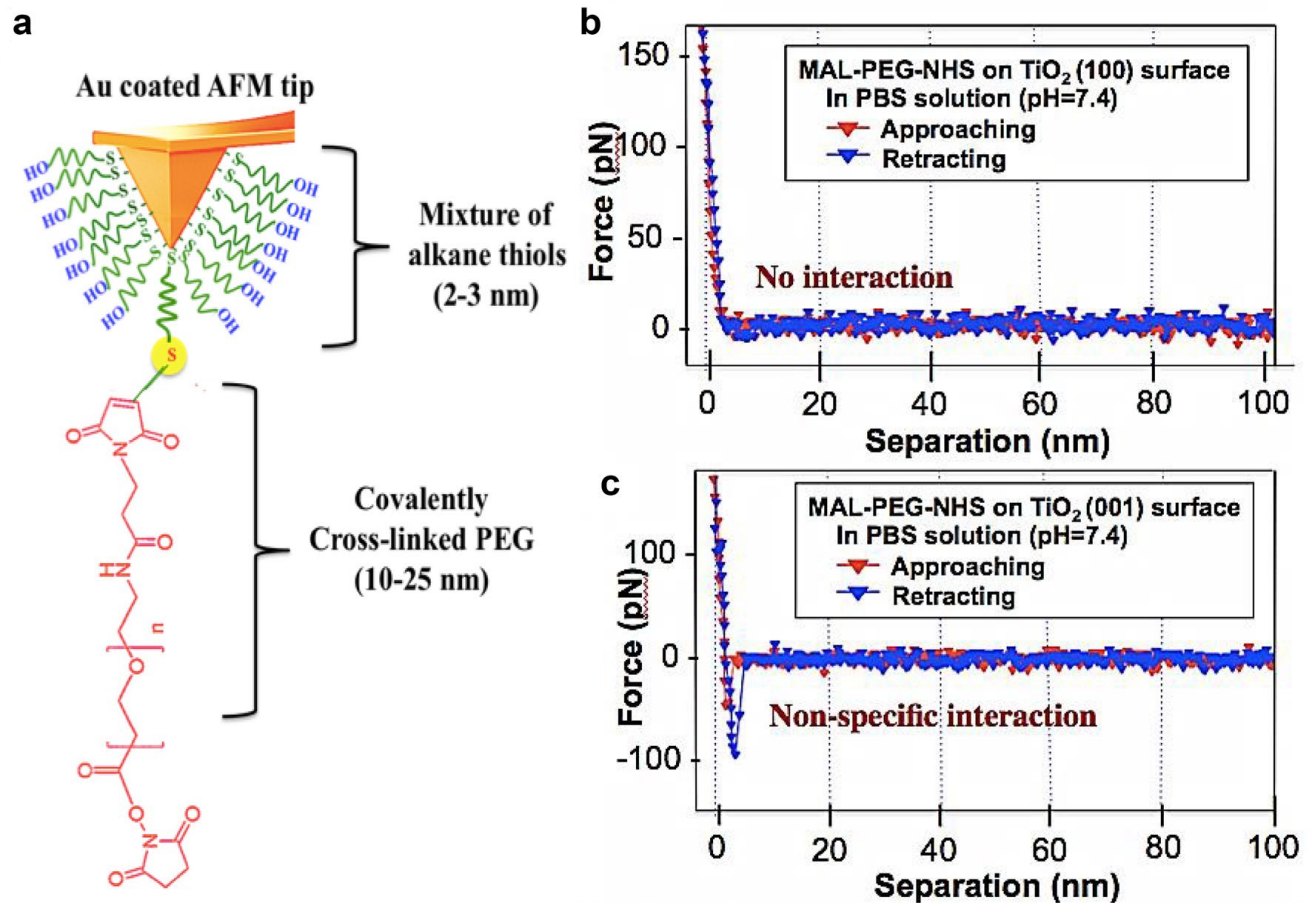
(**a**) Schematic presentation of the modified AFM tip that was used in this experiment; Representative force curves of PEG-modified tip (**b**) on TiO_2_ (100) and (**c**) on TiO_2_ (001) surface

In order to explain the difference between the force curves, we conducted further experiments to check the interaction between the PEG cross-linker and TiO_2_ (110), (100), and (001) substrates. The tip modification process was repeated without the last step, i.e., without the attachment of amino acid molecules. In other words, the AFM tip modification was stopped after the attachment of the PEG cross-linker (Fig. 7a). Measurements were performed in PBS (pH 7.4) solution under the exact same conditions (loading rate, loading force, etc.) of the glycine experiment.

Force measurements did not reveal any specific interaction of PEG-modified tips with TiO_2_ (100) and (001) surfaces. Measurements on the TiO_2_ (100) surface were dominated by force curves displaying no interaction (Fig. 7b), while some curves showing non-specific interaction were also observed. When we changed the substrate to TiO_2_ (001), only force curves showing non-specific interactions caused by electrostatic forces were obtained (Fig. 7c).

**Fig. 8:**
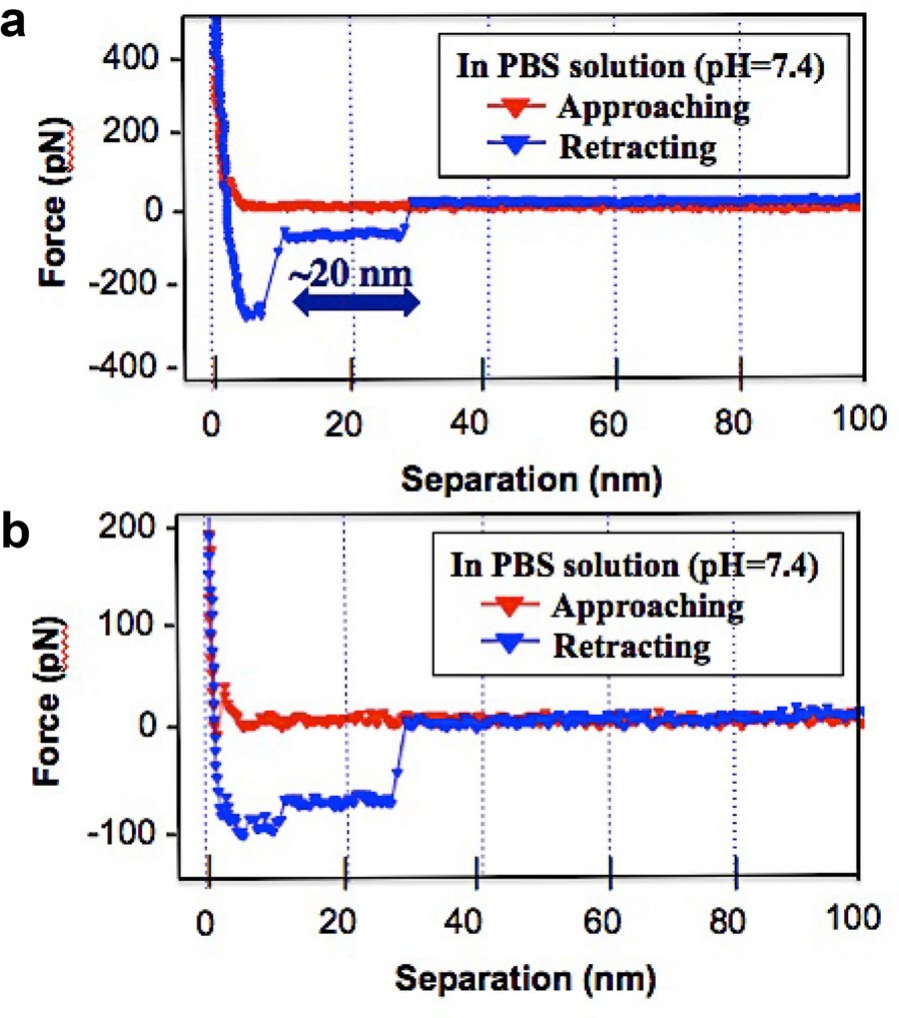
**a**, **b** Representative force curves between PEG-modified tips and the TiO_2_ (110) surface

Interestingly, a different pattern of interaction was obtained in the case of the TiO_2_ (110) surface. We observed a plateau in the force measurements between the PEG-modified tip and the TiO_2_ (110) surface (Fig. 8a, b). This plateau represents a weak physical adsorption of polymer molecules or binding with the surface via the formation of ionic bonds. The length of the plateau directly reflects the length of the adsorbed polymer, whereas its height corresponds to the desorption force that is required to desorb one or multiple polymers from the opposite surface [16].

The average plateau length observed in this experiment was around 20 nm, which corresponds to a considerable extension of the PEG molecule. However, we cannot be certain whether one or multiple polymer chains are involved in this plateau feature. Plateau force curves in AFM studies have no clear interpretation, thus it is difficult to conclude whether we are dealing with a single polymer chain interaction. In rare occasions, force curves displaying some overstretching and step-like plateaus were obtained. These steps represent the desorption of individual molecules of different lengths. Such weak long-range attraction indicates that some kind of contact is maintained over distances exceeding the standard length of the PEG molecule.

Most importantly, this plateau feature was only detected when measuring the TiO_2_ (110) surface, implying that it has unique features of adsorption towards glycine molecules compared to TiO_2_ (001) and (100) surfaces. Our force measurement result was indeed correlated with the difference that was observed between the force curves of glycine on different TiO_2_ surfaces.

As a reference, we performed force measurements on the TiO_2_ surfaces using tips that had only undergone the first step of modification (i.e., they had only been modified with the thiol mixture). Only force curves showing molecular aggregation or non-specific interactions due to van der Waals attraction forces were obtained. This proves that the plateau force of interaction indeed originated from the molecules on the tip and the substrate.

### Glycine adsorption mode on TiO_2_ (110) surface

There are plenty of previous studies about glycine adsorption on TiO_2_ crystals. Most of them used theoretical approaches to determine the binding model of glycine on TiO_2_. According to literature based on DFT molecular dynamics simulations, glycine is adsorbed on TiO_2_ via its zwitterion form, preferentially via deprotonation of the carboxyl group, and forms a (2 × 1) overlayer [3]. Ojamäe et al. suggested a binding model in which the adsorbate binds to surface titan atoms through its carboxylic end (by the formation of an oxygen bridge) and to a surface oxygen ion through its ammonium end (by an H–bond).

In this model, hydrogen bonding via the amine group leads to the further stabilization of the adsorption. They conclude that even though the bridge and monodentate modes are highly favorable, all of these binding modes can contribute to the glycine adsorption on the TiO_2_ (110) surface. The bidentate modes, where the two oxygen atoms in the carboxylic group attach directly to the fivefold-coordinated Ti sites at the surface, are analogous to the most favorable adsorption mode of formic acid described above. Glycine can also form hydrogen bonds with the surface via the amino group, providing additional stability and leading to a tilting of the molecule with respect to its “vertical” configuration [6].

Moreover, Hazen et al. reported that the TiO_2_ (110) surface consists of steps at the atomic scale. The remarkably uniform pattern of steps has an orientation that provides under-coordinated Ti atoms to adsorb amino acids. They strongly suggested that amino acids attach at these steps on the (110) surfaces of rutile. It is known that, in all crystals, steps present special bonding environments for adsorbing molecules.

## Conclusions

In this work, we studied the interaction between glycine and three different TiO_2_ crystal surfaces for the first time at the molecular level using AFM force techniques. The adsorption of glycine on TiO_2_ was observed by AFM force analysis in which all three crystals [TiO_2_ (110), (001), and (100)] used in this study showed specific interactions with the glycine-modified AFM tips, whereas no adhesion was observed on pyrite. The surface force results were in good agreement with the TDS measurements in which glycine showed a desorption peak on the TiO_2_ surfaces, whereas no desorption was observed on mica and pyrite.

The difference in adsorption on the TiO_2_ surfaces was attributed to the shape of force curves on the TiO_2_ (110) surface. The adhesion forces and the shapes of the force curves on TiO_2_ (001) and (100) surfaces were similar. However, the TiO_2_ (110) surface showed a different feature of adhesion in both the AFM force results as well as in the TDS measurements. We assume that the PEG cross-linker may interact with the TiO_2_ (110) surface before the glycine adsorption. Therefore, the crystal orientation of TiO_2_ may influence the amino acid adsorption pathway; however, how the surface structure of this crystal attaches the organic molecules is still under investigation.

As outlined in the introduction, the biomolecule adsorption on TiO_2_ surfaces is also studied for use in many technological and medical applications. In origins of life studies, a fundamental level of understanding of the role of minerals in chemical evolution can be achieved by studying how molecules interact with TiO_2_ surfaces in a way similar to that applied to other candidate catalysts. Our results may contribute to the chemical evolution studies by adding to the knowledge of how biomolecules interact with the TiO_2_ surface, which is considered as one of the host inorganic catalysts in prebiotic evolution studies. Moreover, knowledge of these interactions might also facilitate the use of TiO_2_ in biotechnological applications.
